# Understanding determinants of acute stroke thrombolysis using the tailored implementation for chronic diseases framework: a qualitative study

**DOI:** 10.1186/s12913-019-4012-6

**Published:** 2019-03-20

**Authors:** Lesli E. Skolarus, Gina M. Neshewat, Lacey Evans, Molly Green, Narmeen Rehman, Zach Landis-Lewis, Jillian Welsh Schrader, Anne E. Sales

**Affiliations:** 1 Stroke Program, Department of Neurology, University of Michigan, 1500 E Medical Center Dr, SPC 5856, Ann Arbor, MI 48109-5856 USA; 2 School of Public Health, University of Michigan, Ann Arbor, MI USA; 3 Department of Learning Health Science, University of Michigan, Ann Arbor, USA; 4 Center for Clinical Management Research, Veterans Affairs Ann Arbor Healthcare System, Ann Arbor, MI USA; 5 Hurley Medical Center, Flint, USA

**Keywords:** Stroke, Implementation science, Thrombolysis, Health disparities

## Abstract

**Background:**

The Tailored Implementation in Chronic Disease (TICD) framework is a comprehensive framework describing the determinants of implementation success that has been used extensively in primary care settings. We explored the utility of the TICD to identify determinants of practice in an acute setting, namely guideline concordant acute stroke thrombolysis in a low-resourced, predominately minority serving, large, Emergency Department (ED).

**Methods:**

Through workshops and expert review, we developed an interview guide informed by the TICD framework. We then conducted semi-structured interviews with data collected through written transcripts, audio transcripts or interviewer notes based on participant availability. Three independent coders then performed a content analysis using template analysis, but open to new determinants that arose from the data, into the TICD framework.

**Results:**

We performed a total of 15 semi-structured interviews with ED acute stroke providers including medical technicians, nurses, and physicians. We found that guideline factors, individual health professional factors, and patient factors domains were barriers to guideline concordant acute stroke thrombolysis. The domain professional interactions was a facilitator to treatment. We identified three determinants, healthcare professional burnout, health care professional turnover and surrogate decision making, that are not part of the TICD framework.

**Conclusions:**

Most determinants of acute stroke thrombolysis are included within the TICD framework. Inclusion of healthcare professional burnout, healthcare professional turnover and surrogate decision making may assist in expanding the TICD to time-sensitive ED conditions. Further work is needed to confirm this finding and to establish whether the TICD is applicable for use in non-time sensitive ED conditions. Interventions that address guideline, individual health professional and patient factors may improve guideline concordant acute stroke thrombolysis.

**Electronic supplementary material:**

The online version of this article (10.1186/s12913-019-4012-6) contains supplementary material, which is available to authorized users.

## Background

Implementation frameworks guide the understanding of the determinants of evidence- based practices [1]. The Tailored Implementation in Chronic Disease (TICD) framework is one such framework. Developed by Flottorp et al., [2] it is a comprehensive framework based on an extensive systematic review of determinants of change in primary and secondary care as well as public health services. The TICD explores determinants of practice in seven domains which include guideline factors, health professional factors, patient factors, professional interactions, incentives and resources, capacity for organizational change, and social, political, and legal factors [2]. The TICD has been used extensively in primary care for patients with chronic disease [1] and recently in a systematic review of acute stroke care [3].

A cornerstone of acute stroke care is acute stroke thrombolysis via a medication called Tissue Plasminogen Activator (tPA). tPA reduces post-stroke disability. It must be administered in the Emergency Department (ED) within 4.5 h of stroke symptom onset — earlier treatment results in a significantly greater chance of stroke recovery (i.e. Time is brain) [4]. Once the stroke patient arrives to the hospital, a multi-step, multi-provider process is emergently initiated with an administration goal of acute stroke treatment within 60 min of the stroke patient arriving to the hospital — faster times suggest a greater optimization of the multi-step processes and lead to better patient outcomes. Despite strong evidence of efficacy and effectiveness, as well as strong guideline endorsement from neurologic associations [5, 6] acute stroke thrombolysis remains an underutilized treatment [7]. Notably ED guidelines have a weaker endorsement of acute stroke thrombolysis than the neurology guidelines and there are a vocal group of ED physician opponents [8, 9].

Overall, little attention has focused on understanding guideline concordant provision of acute stroke thrombolysis in the ED [10]. Recent systematic reviews of determinants of guideline concordant acute stroke care categorized determinants into the Theoretical Domains Framework (TDF) or the TICD framework [3, 10] Because implementation research frameworks were not used in the primary studies, these secondary analyses were limited by a lack of contextual details. [3, 10]. In this context, we explored the use of the TICD framework to understand the determinants of guideline concordant acute stroke thrombolysis in an urban, under-resourced, predominately minority-serving ED. Results of this study will inform others on the use of the TICD when exploring determinants of an acute event in an acute setting and will enhance the understanding of guideline concordant acute stroke thrombolysis.

## Methods

### Study design

We performed a qualitative study using semi-structured interviews, informed by the TICD, to identify which determinants are factors that obstruct or enable delivery of guideline concordant acute stroke thrombolysis [11]. Determinants are sometimes referred to as barriers/enablers, barriers/facilitators or problems and incentives [11]. Interviews were conducted with hospital providers of stroke thrombolysis at an ED.

#### Ethics, consent and permissions

This study was determined not regulated as human subjects research by the Institutional Review Board of the University of Michigan and the collaborating hospital’s IRB committee. All participants were verbally consented, which was approved by the University of Michigan and the collaborating hospital’s IRB committee. Verbal consent was obtained to retain anonymity and because this is a no more than minimal risk study.

#### Developing the interview guide

Using implementation frameworks to identify determinants influencing practice has the advantage of enhancing understanding of the complete system; and after intervention implementation, to understand whether and how the intervention improved guideline concordant care in a specific clinical context [12]. To begin, we developed an interview guide and coding framework based on the TICD. Because the TICD contains 57 possible constructs within seven domains, we began with two workshops, one at an Academic Comprehensive Stroke Center and the other at a Primary Stroke Center, to better focus the semi-structured interviews [11]. The workshops, which lasted about 90 min, were attended by vascular neurologists, ED physicians, ED and neurology nurses, stroke program directors and the research team. Ultimately, we decided to forego questions from the social, political and legal factors domain to be mindful of participants’ time. The interview guide was then drafted by a vascular neurologist (LS) with a background in qualitative research [13–15] with the support of an implementation researcher (AS). The interview began with an introduction and rapport building question and then proceeded into the TICD domains. The first section focused on individual health factors, followed by guideline factors, and capacity for organizational change. Questions pertaining to professional interactions, incentives and resources, and patient factors were also included in these sections (see Additional file 1).

### Participants

We conducted interviews between March and July 2017. We undertook a purposive sampling of providers who oversee or deliver direct patient care to acute stroke patients. Potential participants were identified to reflect various roles in clinical practice (physicians, nurses, medical technicians), setting (ED and neurology inpatient ward), and leadership defined as manager or director job title (yes and no), to capture a broad range of information about provision of thrombolysis. Interview participants from the purposive sampling pool were requested to provide up to three names of people who they thought may provide additional perspective on thrombolysis administration. These possible participants were approached face-to-face. There were no refusals. We thought these individuals might have divergent opinions about the practice of and potential barriers to acute stroke thrombolysis from those in the original purposive sample [16].

### Procedure

A vascular neurologist (LS—woman) trained in qualitative interviewing, who had no relationship with participants and was not a practicing clinician at the hospital, conducted 15 in-person interviews. The interviewees were aware that the goal of the interviews was to improve stroke care. The interviews took place in a confidential setting within the hospital or their place of work. Initially, the interviewer and two research assistants completed four interviews which the research assistants transcribed in real time. The research assistants then combined their transcripts into one final transcript. Consequently, due to personnel constraints, the interview procedures were changed, and the next eight interviews were recorded and transcribed. We performed a quality check of approximately 10% of the final interview transcripts to verify that the transcription matched the audio recording. Finally, due to the time constraints of three interviewees, participants who provided direct patient care with little autonomy over their schedule, a single interviewer completed the last set of interviews recording detailed notes during the interview. These notes included (a) direct quotations, (b) paraphrased responses, and (c) interviewer identification of patterns or hypotheses [17]. Participants were consented verbally at the start of the interview. Additionally, they were given small tokens of appreciation for participating. Member checks and peer debriefing enhanced credibility [18, 19].

### Analysis

We first de-identified and combined all the transcripts, irrespective of how the data were collected. We then used an online qualitative data management platform, Dedoose, for data management and coding assistance. We performed directed content analysis using the TICD as a template.^18^ Given the TICD has been used predominately in primary care, we were open to new determinants that arose from the data that were not included in the TICD framework. The coding team consisted of three study team members (GN, LE, MG) with expertise in health behavior and health education. Coder-A coded all 15 interviews, while Coder-B and Coder-C coded 8 and 7 interviews, respectively. Two team members independently coded each transcript into the TICD domains. They then routinely met and compared their coding, discussed differences, and agreed on final codes. If an agreement could not be reached between the two independent coders, the 3rd coder was included in the discussion to reconcile a final code.

Information deemed important in understanding guideline concordant provision of tPA for acute stroke, yet did not fit within the TICD framework, was noted. The research team reviewed these excerpts using inductive coding methods and met to achieve consensus on potential additional codes or determinants. Excerpts were used from the transcripts to illustrate each additional code. Professional roles of the participant were also noted.

## Results

### Overview

The interviews were a mean duration of 27 min (SD 6 min) and ranged between 13 and 67 min. Thematic saturation was reached after interviewing 15 participants (ED nursing leadership 2, neurology nursing leadership 3, ED physician 2, vascular neurologist (director) 1, ED nurse 5, and ED medical technicians 2). The three interviews where notes were taken by the single interviewer were shorter in duration and with participants who provided direct patient care with little autonomy over their schedule.

We identified three codes that did not map to TICD determinants. The first inductive code, healthcare professional burnout, is an individual healthcare professional factor. ED leadership noted, **ID002** “*It’s very hard to move the needle in one area because you’re spread out so thin. The ED has a big turn over, the staff population is big, the acuity, the volume of patients is hard, especially for the new nurses, they get burned out early.”* A related inductive code is healthcare professional turnover, which we considered to fall within the incentives and resources domain because of the focus on workforce capacity and financial/non-financial incentives. A nurse noted, **ID001** *“We have expanded a lot in the past few years. When we opened up in xx and until now, we have added probably 70 or more FTEs. When you have such a large group you do have a lot of turn over. For example, even a 5% turnover is a lot with as many staff as I have. We have senior nurses who know the protocol in and out, but we always bring it for the new staff.”* Finally, the third code, surrogate decision makers, was included in the patient factors domain. One nurse noted, **ID011** *“My last stroke code was a patient with arm weakness. We were initially told by EMS that it started one hour prior to coming to the hospital but when the patient’s daughter came she reported it had been going on for hours. Neither the patient or his daughter recognized it as a stroke.”* A nurse manager noted the importance of the pre-administration tPA discussion, **ID001** “*I think the key is the education piece, when you have a great provider that gives great education, the family or the patient understands, they want the medicine*.” One physician noted, **ID015** *“this [tPA administration] will always go down to how people process risk, you know, people’s emotional attachment to those decisions…that goes for both patients, their families, and for physicians.”*

### Guideline factors

We found that barriers were more common than facilitators within the guideline factors domain (Table 1). Physicians were skeptical about the benefits of administering tPA. One physician noted ambiguity of benefit, **ID015** “*My general take on the literature is that, at least as far as tPA goes, is that there’s probably a subset of patients who are likely to benefit. I’m still not entirely clear from the literature, who that is, which makes it a little harder to predict how your patient will benefit.”* The concerns about the strength of the recommendation and quality of evidence supporting the recommendation potentially lead to an overemphasis of the risks of tPA during discussions with patients and families. One physician noted, **ID013** *“[physicians] oversell the risks or they can minimize benefits”.* Meanwhile, nurses and medical technicians had less concerns about the strength of the recommendation, but overwhelmingly noted that effort and compatibility were significant barriers to tPA administration, particularly due to frequent neurologic checks and charting. One nurse noted, **ID011** “*tPA takes time away from our other patients. We have to go to 1:1 and thus someone needs to cover our other patients. tPA is one hour of excitement and then three hours of charting afterwards. The neuro-checks are really a lot of work.*” Clarity and identification of eligible stroke patients was noted as a barrier with regards to patients with relative or minor exclusions such as those with improving stroke deficits. Participants frequently mentioned observability as a barrier. This construct highlighted the disconnect between the ED and neurology floor providers since stroke patients typically present at their most severe in the ED and then improve over time in another hospital setting. Therefore, the ED providers are uninformed about the patient’s outcomes while the floor providers are uninformed about the patient’s initial stroke presentation. One nurse noted, **ID001** “*We never know if we actually helped them.”* Participants reported accessibility of the intervention and recommendations, such as the presence of stroke protocols in the ED, and trialability, such as mock stroke codes, were facilitators.

**Table 1:** Barriers and Facilitators to Guideline Concordant Acute Stroke Treatment described via a Tailored Implementation in Chronic Disease (TICD) framework analysis

TICD Domain	Barrier or Facilitator	Construct
Guideline Factors	Barrier	Strength of recommendation
		Quality of Evidence Supporting the Recommendation
		Effort
		Clarity
		Observability
		Compatibility
	Facilitator	Accessibility of the Intervention
		Accessibility of the Recommendation
		Trialability
Individual Healthcare Professional Factors	Barrier	Emotions
		Intention and motivation
		Self-monitoring or feedback
		Agreement with the Recommendation
		Domain Knowledge
		Health care professional burnout*
	Facilitator	Self-Efficacy
		Awareness & familiarity with the recommendation
		Expected Outcome
Patient Factors	Barrier	Patient needs
		Patient behavior
		Patient beliefs and knowledge
		Patient preferences
		Surrogate decision makers*
Professional Interactions	Barrier	Referral processes
	Facilitator	Communication & Influence
Incentives and Resources	Barrier	Availability of necessary resources
		Health care professional turnover*
	Facilitator	Quality Assurance & Patient Safety Systems
		Assistance for Clinicians
		Continuing Education System
Capacity for Organizational Change	Barrier	Monitoring and feedback
		Regulations, rules, policies
	Facilitator	Capable Leadership
Guideline Factors	Barrier	Strength of recommendation
		Quality of Evidence Supporting the Recommendation
		Effort
		Clarity
		Observability
		Compatibility
	Facilitator	Accessibility of the Intervention
		Accessibility of the Recommendation
		Trialability
Individual Healthcare Professional Factors	Barrier	Emotions
		Intention and motivation
		Self-monitoring or feedback
		Agreement with the Recommendation
		Domain Knowledge
		Health care professional burnout*
	Facilitator	Self-Efficacy
		Awareness & familiarity with the recommendation
		Expected Outcome
Patient Factors	Barrier	Patient needs
		Patient behavior
		Patient beliefs and knowledge
		Patient preferences
		Surrogate decision makers*
Professional Interactions	Barrier	Referral processes
	Facilitator	Communication & Influence
Incentives and Resources	Barrier	Availability of necessary resources
		Health care professional turnover*
	Facilitator	Quality Assurance & Patient Safety Systems
		Assistance for Clinicians
		Continuing Education System
Capacity for Organizational Change	Barrier	Monitoring and feedback
		Regulations, rules, policies
	Facilitator	Capable Leadership

*Inductive Codes

### Individual health professional factors

We found that individual health professional factors were also dominated by barriers. Emotions such as fear, from the infrequency of the treatment and fear of complications, were noted. One nurse noted, **ID005** “*I think it’s tPA because it is not given on a daily basis. They worry about the risk because they know what could happen to the patient, so it’s all a scary thing for staff members and they want to make sure… the scary part is making sure that they are doing correctly.*” Physicians were more concerned with the bleeding risk, **ID015** “*to the extent that I can speak for a lot of emergency physicians, the fear of having something awful happen sometimes outweighs even the very rational, informed, educated understanding of what the literature says about the likelihood of patients having less disability in the future*.” Knowledge, particularly surrounding minor strokes, was a barrier. One nurse noted, **ID 012** “*The subtle ones are the hardest. They are hard on the patient, hard on the nurses and even the doctors.”* Motivation and intention was a barrier mostly centered around medical documentation. Self-monitoring or feedback was a barrier. We found that feedback reports were consistently distributed to the leadership but rarely distributed to the direct care providers. Participants were interested in process and outcome level feedback, as one medical technician noted, ***ID008*** *“cause if I get that feedback then I know if there’s an area that I would need to improve upon or to help with the patient’s outcome*.” However, there were concerns from nursing leadership that the staff is *‘at information overload.’*

There were differences of opinion regarding agreement with the recommendation to deliver tPA. Overall, nurses and technicians were more in favor of tPA treatment whereas physicians were more skeptical. One physician noted, **ID013** *“There is definitely a vocal minority of ER physicians that are tPA skeptics and non-believers and they definitely exist in the community, and I don’t think that, there’s probably a little of influence of that up here, but I don’t think it really, there’s no one in our group that’s not offering tPA but I know that exists out there.”*

Self-efficacy, awareness and familiarity with the recommendation, and expected outcomes were generally weak facilitators. Overall, providers felt confident enacting the acute stroke protocol. Yet, less self-efficacy was noted diagnosing a stroke and providing care to severe stroke patients post-tPA. While many providers had not reviewed the guidelines, all providers were familiar with the acute stroke treatment protocol. Despite most providers agreeing that administering tPA would lead to better outcomes for appropriate patients, there were some providers who were not convinced that tPA improves outcomes. On physician noted **ID 015** *‘and then there’s people who are kind of like skeptics--based on their understanding of the literature, their own patient care experiences, thought skeptics as a lot of emergency physicians are, based on a combination of bad personal experiences with maybe a little superstition too.’* Expected outcomes were sometimes discussed as a barrier when interviewees observed a negative patient outcome with tPA. One nurse noted, **ID006** *“He [patient who received tPA] ended up getting intubated and had to go to the Neuro-ICU… I remember that being my first experience [with tPA] and being like oh my gosh, you know, why do we push tPA if it can have this kind of outcome.”* However, there were also personal endorsements of tPA, nurse **ID001** shared**,** *“If I had deficiencies would I want tPA, yes. I believe it works well enough to take the chance. I believe it does.”*

### Patient factors

Patient factors were perceived to be barriers to thrombolytic treatment. Many providers noted inadequacy of stroke preparedness as a barrier to thrombolytic treatment. Doctor-patient communication needs was also noted as a barrier. A nurse noted, **ID0012**., “*Yes [patients would want to receive tPA], if they fully understand. You have to remember that most people are only at a 4th-5th grade level. Sometimes patients do not understand the doctors, especially the residents. Residents do not know how to talk to the patients. I can tell when people are not understanding as they do not ask any questions—that is bad. Physicians need to be sure patients understand.”* Similarly, patient preferences surrounding patient-provider communication was a barrier that at times led to tPA refusals or delays in care. One physician noted, **ID013** *“A couple people have refused it, but not too many, why? I think some patients have like a skepticism of medicine and then when it’s presented they think it’s something experimental.”* Another nurse noted, **ID012** *“I think we give too much warning with tPA….. I think we tell patients about all the bleeding and that they may get worse or die and then patients get scared. I mean, why do we focus so much on the risks. This is nothing like MI [Myocardial Infarction] when we just take them to the cath lab. Or when we stop and restart someone’s heart. We do not tell the patients all this warning, we just do it. There is no decision. But for stroke we talk too much about all the warnings which scares the patient. We should not give them so many choices. The stroke will be with them for the rest of their life.”*

### Professional interactions

Communication and influence was a notable facilitator. Participants noted strong, respected opinion leaders, at one point describing *‘the [Drs. Name and opinion leader] phenomenon*,’ where he/she was quoted as ‘*this is what we need to do, tPA this is your best friend, this is your medical legal best friend’.* There was perceived excellent physician-nurse communication; communication and support among nurses was a notable strength. One nurse described the support after a case that did not go well, **ID006** “*I was like oh my gosh I never want to give tPA again and another nurse came up to me and he was like ‘dude, it’s one patient, you’re gonna have other patients and it’s gonna be positive outcomes and it’s gonna be worth it.’ So, that was kind of my enlightening. I was kinda like well I can’t be afraid to give this anymore.”*

The referral process was a minor perceived barrier due to the large number of EMS agencies that serve the hospital, but overall there was a sense of strong communication and oversight. The changes in the patient’s history from EMS to ED was noted but was generally understood as a function of EMS time pressures that preclude detailed history taking at the scene.

### Incentives and resources

Availability of necessary resources was a barrier noted by many participants. Individuals cited the ED and hospital are often full and the ED is commonly used as a primary care clinic in the city. The triage system was noted to be effective because providers can assist in stroke codes. Quality assurance, assistance for clinicians, and continuing education systems were all facilitators. Nurse **ID005** noted, “*I think [the quality meeting] really has [been helpful],.., if there is an issue we come together and figure out how to fix those issues*.”

### Capacity for organizational change

Monitoring and feedback was a perceived barrier. Feedback was provided to the leadership but not disseminated to providers. When asked about whether they would want to receive feedback one medical technician noted, **ID009** *“I think it would help us improve, you know on how would I put it, our self-awareness of, okay well we did a good job, we recognized that this was something serious.”* The ED leadership, particularly nursing noted competing information interests and time constraints. **ID001** shared, *“Our staff has had information overload. Sometimes we have a shift huddle, it can go on for 15 minutes of blah blah blah, if you have 15 emails do you really want to look at your [stroke code report]? How do I make that important, how do I get that to them, and (the) least important out of the way? I don’t think we have the time.”* Rules, regulations and policies centered around the number of EMS agencies. Capable leadership was seen as a facilitator. **ID002** *“[leader name] has been a key to all this.”*

## Discussion

This qualitative study of healthcare providers in an urban, under-resourced, predominately minority serving ED used the TICD to identify determinants influencing guideline concordant acute stroke thrombolytic treatment. It is one of the first to use the TICD for guiding data collection, analysis, and interpretation in an acute setting and disease process. [3, 10] While most determinants were included in the TICD, inclusion of healthcare professional turnover, healthcare professional burnout, and surrogate decision making may help to expand the TICD to the acute setting. Three domains, guideline factors, individual health professional factors, and patient factors were primarily barriers to thrombolysis treatment. The domains professional interactions and determinants of capable leadership, and quality assurance were facilitators to guideline concordant acute stroke thrombolytic treatment.

Much of the work in optimizing guideline concordant acute stroke thrombolysis has focused on optimizing clinical pathways without taking into consideration the health care provider, patient and system within which these pathways operate [20]. Our more holistic approach, using the TICD framework, found that clinical care pathways were facilitators and offers greater insight into how these pathways operate. We found that overall the TICD framework is applicable to care processes outside of chronic illnesses in the primary care setting. We were able to code most of the data into the TICD with the exception of the need to include three inductive codes: healthcare professional burnout, health care professional turnover, and surrogate decision making. Given the pace and the acuity of the ED, it is not surprising that burnout, which may result in healthcare professional turnover, would be a barrier. Prior studies have confirmed our findings and recommend careful consideration of the role of ED physician turnover in cluster randomized trials is recommended [21, 22]. Furthermore, unlike chronic disease patients, many acute stroke patients lose decision making capacity. In fact, over 35% of acute stroke patients have aphasia or cognitive impairment requiring a surrogate decision maker [23]. Further research is needed to confirm these new determinants as a way to expand the TICD framework into acute settings and time sensitive guideline concordant treatment.

Among patients who are eligible for acute stroke thrombolytics, tPA should be delivered to the vast majority of patients [24]. Previously researchers have found about 7% of eligible acute stroke patients refuse tPA [25]. Our findings provide insight as to why patient’s might refuse or delay tPA administration. Physicians were skeptical of the benefits of thrombolysis and had concerns with the strength and agreement with the recommendation. This uncertainty may result in over-emphasis of the risks and minimization of the benefits of acute stroke thrombolysis. Additionally, patients and families may not understand the risks and benefits of acute stroke thrombolysis preventing informed decision making. Improving physicians beliefs in the acute stroke thrombolysis guidelines and optimizing patient-doctor communication may increase acute stroke thrombolysis treatment rates, decrease thrombolysis treatment times and reduce racial differences in acute stroke thrombolysis treatment rates [25].

Organizational level barriers were identified. Although feedback was perceived as an individual facilitator, feedback reports were not disseminated to front-line providers. One notable explanation for the lack of dissemination was the perception of competing priorities and information overload. Developing mechanisms to optimize an organization’s opportunities to benefit from audit and feedback based on need and aptitude for improvement would be helpful.

Facilitators to acute stroke thrombolytic treatment were identified. The acute stroke thrombolytic guidelines and protocols were readily accessible. Communication among and between providers was a notable strength. The hospital has strong leaders who are highly respected and supportive of tPA treatment. Similar to a Swedish study, [25] we also found quality assurance committee and individual feedback reports were perceived as favorable.

Three different strategies for data collection were used: 1) audio recorded transcripts; 2) written transcripts; and 3) interviewer notetaking. Most of the data was obtained through audio recording and transcriptions. We felt there was little to no quality difference between written and audiotaped transcripts. We conclude the presence of two-real time transcriptionists is feasible and time-saving for longer interviews but nearly equivalent for shorter interviews. The interviewer notetaking required only one member of the research team, but had some limitations. Direct quotes were more difficult to obtain and only the most salient data was documented verbatim. The interviewer notetaking interviews were conducted last so the interviewer had a sense of the hospitals acute stroke thrombolysis processes which assisted the data collection. If interviewer notetaking were to be the primary method of data collection, data collection instruments should be considered, [17] or used in combination with audio-recording if needed [26].

This study had limitations. First, this is a single center study and the TICD determinants are likely to be context specific. Further research across sites to confirm our findings is needed. The interviewer was a vascular neurologist, although not a practicing clinician where the interviews took place and with no prior relationship with the participants, we cannot exclude that the mere fact the interviewer was a vascular neurologist influenced the results. Previously we attempted an interview with a non-medically trained interviewer but found that content and richness of data was lost due to miscommunication surrounding medical terminology. Patients were not interviewed and thus findings are limited to the provider’s perspective of patients and not the patients’ own perspective.

## Conclusion

In summary, exploration of the determinants of guideline concordant acute stroke thrombolysis can be performed using the TICD framework. Future work to expand the TICD to the time-sensitive acute setting by including healthcare professional burnout, healthcare professional turnover, and surrogate should be considered. In addition, further work is needed to establish whether the TICD is applicable for use in non-time sensitive ED conditions. Interventions that address guideline, individual health professional and patient factors may improve guidelines concordant acute stroke thrombolysis.

## Additional file

Additional file 1: Interview guide exploring guideline concordant acute stroke thrombolysis. Interview guide. Qualitative interview guide. (DOC 94 kb)

## Abbreviations

ED: emergency department
TICD: tailored implementation in chronic disease
tPA: tissue plasminogen activator
