# Supplementary material for: Understanding determinants of acute stroke thrombolysis using the tailored implementation for chronic diseases framework: a qualitative study
Source: BMC Health Serv Res. 2019 Mar 20;19:182. doi: 10.1186/s12913-019-4012-6 (PMC6425596; doi:10.1186/s12913-019-4012-6)
Supplement: Supplementary file 1 — Interview guide exploring guideline concordant acute stroke thrombolysis. Interview guide. Qualitative interview guide. (DOC 94 kb) [file 12913_2019_4012_MOESM1_ESM.doc]

**Interview Guide: Hospital Staff**

**Introduction**

*Hello I am [your name] from the Neurology Department at the University of Michigan Health System. We have partnered with (xx) to work together to improve acute stroke care. Before we begin I want to let you know that we are interested in your comfort in this interview and we are here to talk about anything you would like to talk about.*

Date/ Time:

Hospital staff position:

Transcriber:

Respondent ID:

| **PART 1**  **Construct/Concept: Warmup / Rapport Building**  **Questions** |
| --- |
| 1. **Could you begin by telling me a little about your role, and how long you have worked, in the (xx) Emergency Department**?   *Type response here* |
| **PART 2**  **Construct/Concept: tPA administration, Facilitators and Barriers**  **Questions** |
| **In this section of the interview, we will mainly focus on what happens during a code-180 and tPA administration from your experience as a {position of the interviewee} at (xx).** |
| 1. **Tell me about a time when you were involved in a stroke code.**   *Type response here*  **Probes**:   - When is the stroke code activated? - How are stroke codes prioritized compared to other areas in the ED? *Type response here*   - In general, how feasible is it to administer tPA? *Type response here* |
| 1. **Describe the communication process within the hospital during a stroke code when administering tPA?**   *Type response here*  **Probes**:   - - What works particularly well? What may improve its administration? *Type response here*   - What changes would benefit interactions with systems outside the hospital, such as EMS, to be able to implement tPA treatment more effectively? *Type response here* |
| 1. **What makes it easier to give tPA**?   *Type response here*  **Probes**:   - In what ways do team members interact and communicate that facilitate (or hinder) adherence to tPA? *Type response here* - What kinds of resources would allow for increased use of tPA, (personnel, easier access to head CT, meds)? *Type response here* |
| 1. **What might be preventing patients from receiving tPA more**?   *Type response here*  **Probes**:   - Describe the barriers that may be hindering tPA treatment? *Type response here* - Examples may include: delayed pt recognition, EMS prenotification, delayed activation of code-180, slow labs, slow head CT, slow pharmacy, availability of neurology. *Type response here* |
| 1. **Do you have the assistance needed (checklists, patient information, decision aids and support, clinical supervision) to administer tPA**?   *Type response here* |
| 1. **Do you think that tPA treatment leads to better outcomes?**   *Type response here*  **Probes**:   - - Do you think patients want to receive tPA? If so, why? *Type response here* |
| 1. **Is there someone whose opinion you look up to that affects how you perceive the use of tPA?**   *Type response here*  Probes:   - - How does leadership play a role in tPA administration? *Type response here* |
| 1. **Is there a way that the electronic system is helping you to administer tPA**?   *Type response here* |
| **PART 3**  **Construct/Concept: Protocols**  **Questions** |
| **In the first part of the interview we discussed tPA administration. This next section of the interview is about your experience with stroke protocols.** |
| 1. **Do {position of the interviewee} in your group/area use acute stroke protocols**?   *Type response here*  **Probes**:   - Where do you find the protocols? *Type response here* - Walk me through a situation where you used the acute stroke protocols? *Type response here* - How feasible is it to use the protocols in your practice? *Type response here* - How are the stroke protocols consistent with other protocols that you use? *Type response here* - How are the stroke protocols consistent with the current workflow? *Type response here* |
| 1. **How do you and your colleagues view protocols in general? Stroke Protocols**?   *Type response here*  **Probes**:   - Was there a time that you remember when it was not helpful? *Type response here* - What would have made it more helpful? *Type response here* |
| **Regarding your personal experience with the stroke protocols…** |
| 1. **How confident are you in adhering to the protocol**?   *Type response here*  **Probes**:   - On a scale from 0 to 10, with 0 being the inability to adhere to the protocol any of the time, and 10 being the ability to adhere to the protocol all of the time, where is your confidence level to adhere to the stroke protocol? *Type response here* - [If interviewee appears not confident in knowledge of protocol] What type of skills would you like to develop to increase your confidence in following the stroke protocol? *Type response here* - Tell me about a time where you had the protocol and needed more information or knowledge than what was provided to follow it? *Type response here* - Other than additional knowledge and skills about the protocol, is there any else that would increase your adherence to the protocol? *Type response here* |
| **The final questions in this section of the interview pertain to the stroke protocol and its usefulness in the administration of tPA:** |
| 1. **How have protocols improved the administration to tPA?**   *Type response here*  **Probes**:   - How much effort is required to adhere to the protocols to administer tPA? *Type response here* |
| **PART 4**  **Construct/Concept: Guidelines**  **Questions** |
| **Thank you for your responses in the last section. In the next section we will discuss guidelines.** |
| 1. **Do you review the current guidelines**? **🡪** If no, skip to PART 5   *Type response here*  **Probes**:   - From your experience, in what ways do you agree with the guidelines? How do you disagree? *Type response here* |
| 1. **How much of your protocols are based on guidelines**?   *Type response here*  **Probes**:   - What is the quality of evidence supporting the benefits of tPA? *Type response here* |
| 1. **How do you and your colleagues view guidelines**?   *Type response here*  **Probes**:   - What would you say about how well you can rely on the information in the guidelines *(and other sources if they are mentioned)*? *Type response here* |
| 1. **How often are the guidelines updated**?   *Type response here* |
| **PART 5**  **Construct/Concept: Feedback**  **Questions** |
| **Thank you for your responses in the last section. For this next section we will discuss the types of feedback you receive about your performance.** |
| 1. **How much feedback do you receive about your individual performance in acute stroke scenarios**?   *Type response here*  **Probes**:   - - In what ways do you know whether your actions during an acute stroke treatment are mostly appropriate, inappropriate, or mixed? *Type response here*   - Which tasks do you not receive enough feedback about? *Type response here*   - When do you receive this feedback? *Type response here* |
| 1. **When you receive feedback, is it typically verbally, in writing, or other ways**?   *Type response here*   - - What information would you be most interested to receive about your performance as a (clinician/nurse)? *Type response here* - What types of learning opportunities would be most helpful for {position of the interviewee} to learn more about tPA treatment? *Type response here* |
| 1. **How does the existing stroke quality improvement collaborative facilitate increased administration of tPA**?   *Type response here* |
| 1. **Is it possible to try out the acute stroke protocols such as through mock stroke codes**?   *Type response here* |
| **PART 6**  **Construct/Concept: Wrap-up**  **Questions** |
| **With these final questions, our interview will come to a close.** |
| 1. **Is there anything else I need to know to better understand tPA administration in the (xx) ED?**   *Type response here* |
| **Thank you so much for taking the time to discuss your role experience in the (xx). If you have any questions about what we discussed today, or would like to add or change anything at a later time, let us know by contacting…** |
